# Physicochemical Properties of Extracellular Polymeric Substances Produced by Three Bacterial Isolates From Biofouled Reverse Osmosis Membranes

**DOI:** 10.3389/fmicb.2021.668761

**Published:** 2021-07-13

**Authors:** Zahid Ur Rehman, Johannes S. Vrouwenvelder, Pascal E. Saikaly

**Affiliations:** Water Desalination and Reuse Center, King Abdullah University of Science and Technology, Thuwal, Saudi Arabia

**Keywords:** extracellular polymeric substances, biofouling, reverse osmosis membrane, NMR, FTIR, *Bacilli*

## Abstract

This work describes the chemical composition of extracellular polymeric substances (EPS) produced by three bacteria (RO1, RO2, and RO3) isolated from a biofouled reverse osmosis (RO) membrane. We isolated pure cultures of three bacterial strains from a 7-year-old biofouled RO module that was used in a full-scale seawater treatment plant. All the bacterial strains showed similar growth rates, biofilm formation, and produced similar quantities of proteins and polysaccharides. The gel permeation chromatography showed that the EPS produced by all the strains has a high molecular weight; however, the EPS produced by strains RO1 and RO3 showed the highest molecular weight. Fourier Transform Infrared Spectroscopy (FTIR), Proton Nuclear Magnetic Resonance (^1^H NMR), and Carbon NMR (^13^C NMR) were used for a detailed characterization of the EPS. These physicochemical analyses allowed us to identify features of EPS that are important for biofilm formation. FTIR analysis indicated the presence of α-1,4 glycosidic linkages (920 cm^–1^) and amide II (1,550 cm^–1^) in the EPS, the presence of which has been correlated with the fouling potential of bacteria. The presence of α-glycoside linkages was further confirmed by ^13^C NMR analysis. The ^13^C NMR analysis also showed that the EPS produced by these bacteria is chemically similar to foulants obtained from biofouled RO membranes in previous studies. Therefore, our results support the hypothesis that the majority of substances that cause fouling on RO membranes originate from bacteria. Investigation using ^1^H NMR showed that the EPS contained a high abundance of hydrophobic compounds, and these compounds can lead to flux decline in the membrane processes. Genome sequencing of the isolates showed that they represent novel species of bacteria belonging to the genus *Bacillus*. Examination of genomes showed that these bacteria carry carbohydrates-active enzymes that play a role in the production of polysaccharides. Further genomic studies allowed us to identify proteins involved in the biosynthesis of EPS and flagella involved in biofilm formation. These analyses provide a glimpse into the physicochemical properties of EPS found on the RO membrane. This knowledge can be useful in the rational design of biofilm control treatments for the RO membrane.

## Introduction

The formation of biofilms on reverse osmosis (RO) membranes, known as membrane biofouling, is considered a major problem in the water desalination industry. Membrane performance indicators such as feed channel pressure drop, permeate flux and salt passage are negatively affected by accumulation of biofilms (Vrouwenvelder et al., 2010), increasing the operational costs of desalination (Herzberg et al., 2009; Rehman et al., 2020a). Eventually, the biofouled membrane must be replaced early, further increasing the overall cost of water purification.

Biofilm typically consists of microbial cells and extracellular polymeric substances (EPS), comprising of polysaccharides, proteins, DNA, and lipids, which play a crucial role in biofilm formation (Flemming and Wingender, 2010; Nagaraj et al., 2017b). The performance decline in membrane systems is predominantly caused by the EPS, and not by the bacterial cells in the biofilm (Dreszer et al., 2014).

The feed water quality of an RO installation can influence the EPS production (Desmond et al., 2018). Phosphate limitation with biodegradable organic compounds in RO feed water can (compared to higher phosphate concentrations) lead to a higher production of EPS, rapid surface coverage with EPS, and accelerating the increase in feed channel pressure drop (Javier et al., 2020). Phosphate concentration in pretreated RO feed water is typically low (Vrouwenvelder et al., 2010; Javier et al., 2020). For cooling towers fed with phosphate limited water biofilm formation was found, and a higher volume of organic matter per unit of active biomass was compared to no phosphate limited feed water (Pinel et al., 2020).

EPS serves as a matrix that allows microbes to attach and spatially organize on the membrane, while also protecting the microbes from the unfavorable environment (Flemming and Wingender, 2010; Flemming et al., 2016). Polysaccharides and proteins are the major components of biofilm, with polysaccharides being the most abundant (Tsuneda et al., 2003; Zhang et al., 2008). However, the relative amounts of different components of EPS can vary depending on the growth substrate, microbial community, and environmental factors (Ras et al., 2011; Rehman and Rehm, 2013; Rehman et al., 2013; Fagerlund et al., 2016). The composition of EPS affects the viscoelastic properties of the biofilm, such as cohesion, structural integrity, and stress resistance (Lau et al., 2009). Therefore, a thorough knowledge of the composition of EPS produced by bacteria is valuable for guiding measures to mitigate biofouling (Seviour et al., 2019).

The chemical properties of EPS are controlled by functional groups that may be chemically charged (carboxyl and hydroxyl) and polar (aliphatic and aromatic). Moreover, the functional groups play a role in bacterial aggregation and biofilm formation (Hou et al., 2015). The hydrophobic nature of EPS, for example, contributes to the aggregation and formation of flocs (Hou et al., 2015). Also, the hydrophobic functional groups of EPS can interact with calcium ions to form calcium bridges that increase flocculation and membrane fouling (Kim and Jang, 2006). Studies have shown that α-1,4 glycosidic linkages and amide II are well correlated with the fouling potential of bacteria (Maddela et al., 2018). Thus, a better understanding of the chemical properties of EPS, such as the types of bonds and functional groups present, is essential to determine the fouling potential of EPS.

Spectroscopic techniques such as Attenuated Total Reflection-Fourier Transformed Infrared Spectroscopy (ATR-FTIR) and Nuclear Magnetic Resonance (NMR) are well suited to study the chemical structure and chemical nature of organic molecules. These techniques have been extensively used to identify the functional groups, type of chemical bonds, and monomeric composition of organic molecules obtained from diverse environments (Metzger et al., 2009; Abdulla et al., 2010; Jiao et al., 2010; Rehman et al., 2017). Furthermore, high-resolution NMR spectroscopy provides information about analytes at the molecular level with little sample preparation requirements, making it an analytical tool of choice (Alves et al., 2015).

Most studies to date on bacteria isolated from biofouled RO membranes have focused primarily on their ability to form biofilms (Pang et al., 2005; Katebian et al., 2016), whereas studies on the composition and physicochemical properties of EPS are scarce (Nagaraj et al., 2017b). Recently, Nagaraj et al. conducted a chemical analysis of exopolysaccharides produced by bacteria isolated from different locations in a seawater RO treatment plant (Nagaraj et al., 2017b). They identified that monomers such as fucose, rhamnose, and uronic acids were abundant in exopolysaccharides and that they might play a role in biofilm formation (Nagaraj et al., 2017b). However, a detailed understanding of the physical and chemical properties of EPS produced by pure cultures of bacteria isolated from full-scale biofouled RO modules is still lacking.

In this study, we investigated the physicochemical properties of soluble EPS produced by three bacteria isolated from a 7-year-old biofouled RO module harvested from a full-scale RO plant at the end of its life span. In a real-world setting, diverse bacteria colonize the RO membrane and produce the EPS, which is more heterogeneous and complex than pure culture EPS analyzed in this study. Nevertheless, in this study, we investigated the EPS produced by a dominant bacterial genus isolated from the biofouled RO module. This EPS may not be entirely representative of the range of EPS found on biofouled RO membranes. Nonetheless it provides a relevant example of the type of organic matter found on biofouled RO membranes. Various analyses including biofilm formation assay, Size Exclusion Chromatography (SEC), ATR-FTIR, and NMR (both ^1^H and ^13^C) analysis were conducted to identify the physical and chemical features of the EPS that are known to play a role in biofilm formation. Genome sequencing analysis identified genes involved in the production of polysaccharides and the formation of flagella, which play a role in biofilm formation.

## Materials and Methods

### Isolation of Bacteria

The biofouled RO module was harvested from a full-scale desalination plant located on the Red Sea coast in the Kingdom of Saudi Arabia (22.299815 N, 39.116812 E). The water quality parameters and the operational conditions of the plant have been discussed elsewhere (Belila et al., 2016). To isolate the bacteria, the biomass was harvested from the biofouled RO module using a sterile spatula and suspended it in sterile seawater. Serial dilutions of the suspended biomass were made and then 100 μl was plated on Zobell Marine Agar (HiMedia, India, 55.25 g powder/L of Milli-Q) plates, followed by incubation at 30°C for 72 h. The Zobell Marine medium mimics seawater composition and allows bacteria in the marine environment to grow abundantly. Three phenotypically different colonies were selected to be streaked on fresh agar plates (Supplementary Figure 1). This procedure was repeated three times to obtain pure cultures of the selected bacteria. The isolates were then grown in Marine Broth (HiMedia, India), and 500 μl of bacterial culture was mixed with 500 μl of 50% glycerol solution and stored at −80°C for future use.

### Growth Curve Analysis

Pure cultures of bacteria were streaked on Zobell Marine Agar plates and incubated at 30°C overnight. A single colony from these plates was inoculated in Marine Broth and incubated overnight at 30°C with shaking at 120 rpm. These bacterial cultures were diluted to OD_600_ of 0.01 in fresh Marine Broth and incubated at 30°C with shaking at 120 rpm. The OD_600_ of these bacterial samples was measured using a Spectronic 200 (Thermo Fisher Scientific, United States) every hour until the cells reached the stationary phase.

### Biofilm Growth Assay

We performed a biofilm formation assay for the isolates in 96-well tissue culture-treated polystyrene plates (Greiner Bio-One, Germany), as described previously (Barbara and Coffey, 2014). Briefly, the overnight grown cells were diluted to an OD_600_ of 0.05, and 100 μl of the diluted cultures was added to the wells of polystyrene plates under sterile conditions. The plates were incubated for 24 or 48 h and culture media were discarded at the end of each incubation. The plates were rinsed in water and stained with 0.1% of crystal violet (CV) for 10 min. The unbound CV was discarded, and plates were rinsed twice in freshwater and dried overnight. The bound CV was dissolved in 30% acetic acid and the absorption was measured in a microplate reader at OD_590_ using SpectraMax M3 (Molecular Devices, United States).

### Extraction of EPS

A single colony of bacterial isolates was inoculated in Marine Broth using a sterile inoculation loop. The bacterial colony was mixed with vortexing and a volume of 150 μl of this bacterial suspension was spotted on fresh Marine Agar plates and spreaded on an agar surface using a sterile L-shaped spreader. These plates were incubated upside down at 30°C for 72 h. The agar surface was used for EPS extraction as this method mimics biofilm growth and yields the maximum amount of EPS (Rehman et al., 2013). The biomass was scraped from these plates using a sterile spatula and suspended in 1x PBS. The EPS was extracted using formaldehyde and NaOH, as described previously (Liu and Fang, 2002). Briefly, 60 μl of formaldehyde (36.5%) (Thermo Fisher Scientific, United States) was added to 10 ml of EPS solution and incubated at 4°C for 1 h. To this mixture, 4 ml of NaOH (1N) was added and incubated at 4°C for another 3 h. The mixture was centrifuged at 16,000 × g at 4°C for 20 min followed by filtration of supernatant through 0.2 μm syringe filter. The filtrate was dialyzed using 3.5 KDa dialysis membranes (Spectra/Por^®^, United States) at 4°C for 24 h. Finally, the solution was lyophilized at −50°C for 48 hrs.

### Quantification of Polysaccharides and Proteins

The carbohydrate content of EPS was measured using a modified phenol-sulfuric acid method and glucose standards (Dubois et al., 1951). Briefly, the lyophilized EPS was suspended in Milli-Q water at a concentration of 1 mg/ml. We mixed 1 ml of this suspension (and glucose standards separately) with 0.5 ml of phenol, followed by the addition of 2.5 ml of concentrated sulfuric acid. The solution was mixed and allowed it to cool at room temperature for 1 h. The absorbance was measured at OD_485_ using a spectrophotometer (Spectronic 200, Thermo Fisher Scientific, United States).

The protein and DNA content of EPS was measured using a Qubit^TM^ Protein Assay kit and Qubit^TM^ dsDNA BR Assay kit, respectively (Thermo Fisher Scientific, United States). The proteins and polysaccharides were quantified in 1 mg of freeze-dried EPS (total). The amount of protein and polysaccharide reported here represents per mg dry-weight of total EPS.

### Molecular Weight Determination of EPS

We determined the molecular weight of EPS by high-performance gel permeation chromatography (HP-GPC; Agilent Technologies 1260 Infinity) equipped with columns PL-aquagel-OH 60 and PL-aquagel-OH 30 connected in series (length 300 mm and particle size 15 and 8 μm, respectively) (Agilent, United States). The HP-GPC was equipped with a differential refractometer and a UV detector, which was set at 280 nm. For the elution, 0.1 M of NaNO_3_ and NaN_3_ (0.02% w/v) (Honeywell Fluka^TM^, United States) were used. The eluent was degassed and filtered through 0.1 μm pore size membrane filters (MF-Millipore^TM^, United States). The EPS samples were dissolved in eluent at 1 mg/ml concentration and filtered through 0.2 μm filters. A 100 μl of EPS solution was injected into the HP-GPC, flow rate was adjusted to 0.5 ml/min, and the temperature of the columns was maintained at 30°C. The polyethylene glycol standards were used to calibrate the system (EasiVial PEG/PEO, pre-weighted calibration kit, 2 ml, Agilent, United States). The HP-GPC analysis was performed in duplicate.

### Fourier Transform Infrared Spectroscopy (FTIR) Analysis of EPS

We analyzed the lyophilized EPS samples by FTIR spectroscopy in attenuated total reflection (ATR) mode using a Nicolet^TM^ iS10 FTIR spectrometer (Thermo Fisher Scientific, United States) equipped with DTGS detector and SMART iTR^TM^ sampling accessory (Thermo Fisher Scientific, United States). The spectrum was recorded in transmittance mode over a range of 3,900 to 600 cm^–1^ at a resolution of 4 cm^–1^.

### Nuclear Magnetic Resonance (NMR) Analysis of EPS

We performed NMR spectrometry (both ^1^H and ^13^C) to determine the chemical composition of the EPS produced by the RO membrane isolates, as described recently (Khan et al., 2013; Rehman et al., 2017). Briefly, for ^1^H NMR, 2–3 mg of lyophilized EPS was dissolved in 650 μl of deuterated water D_2_O and transferred the solution to 5 mm NMR tubes. The ^1^H NMR spectra was acquired using a Bruker 700 MHz AVANCE III spectrometer equipped with a Bruker CPTCI multinuclear CryoProbe (Bruker BioSpin, Germany). The signal-to-noise ratio was improved by acquiring 1K scans with a recycle delay time of 3 sec. In order to suppress the water peak, each spectrum was induced using an excitation sculpting pulse sequence through a standard program (zgesgp) from the Bruker pulse library. Solid-state ^13^C NMR spectra of EPS were obtained using 900 MHz AVANCE III NMR spectrometer (Bruker BioSpin, Germany) using a triple-resonance 3.2 mm Bruker MAS probe. The temperature was maintained at 25°C. Bruker Topspin 3.5p17 software (Bruker BioSpin, Germany) was used for data collection and processing of spectra.

The total area under each spectrum was calculated for complete ^1^H NMR spectra by trapezoid numerical integration using RStudio (1.2.5001) and trapz function in R-package (caTools) (Kincaid and Cheney, 1991). The relative abundances of the major ^1^H resonances were measured by integrating the area in chemical shift ranges (as previously described by Hertkorn et al. (2013), which were normalized by total chart area.

### Genome Sequencing

The genome sequencing of isolates was done as described previously (Rehman et al., 2020b). Briefly, all the strains were grown in 20 ml of marine broth, centrifuged at 6,000 g for 10 min, and the resulting cell pellet was used for DNA extraction using the DNeasy PowerWater kit (Qiagen, Germany). DNA concentration was measured using the Qubit dsDNA HS/BR Assay kit (Thermo Fisher Scientific, United States). The sequencing library was prepared using NEB Next Ultra II DNA library prep kit for Illumina (New England Biolabs, United States) as per manufacturer instructions. Paired-end sequencing (2 × 301 bp) of the samples was done on Miseq (Illumina, United States) using MiSeq Reagent Kit v3, 600 cycles (Illumina, United States).

### Genome Assembly Annotation

The sequencing reads were trimmed using Cutadapt v. 1.16 (Martin, 2011), and assembled using Megahit v. 1.1.3 (Li et al., 2016). The genome coverage was calculated by mapping reads back to assembly using minimap2 v. 2.12-r827 (Li, 2018). The genomes were annotated using PROKKA v. 1.14-dev (Seemann, 2014), and completeness was calculated using CheckM (Parks et al., 2015). Identification of carbohydrate-active enzymes (CAZymes) was made online using predicted amino acid sequences^1^ (Zhang et al., 2018). The dbCAN2 database was searched for homologous sequences using HMMER with default values (*E* < 1e-15, coverage > 0.35). Comparative genome analysis of RO isolates was done using BLAST Ring Image Generator (BRIG) (Alikhan et al., 2011). For these analyses, the RO2 genome was used as a reference, while RO1 and RO3 genomes were used as a query.

The blastp was used to identify various proteins involved in the production of EPS.

### Data Depositions

The genome sequences reported in this article were deposited to DDBJ/ENA/GenBank under BioProject ID PRJNA616073 and accession number JAAZWB000000000 (RO1), JAAXCU000000000 (RO2), JAAXCV000000000 (RO3). The version described in this paper is version JAAZWB010000000, JAAXCU010000000, and JAAXCV010000000.

## Results and Discussion

Based on colony morphology, three distinct bacterial colonies were selected for further analyses (Supplementary Figure 1). All three isolates showed a lag phase of 2–3 h followed by logarithmic growth for 6 h in Marine growth medium. The bacterial cells entered the stationary phase after 8–9 h of growth (Supplementary Figure 2), which may be due to the depletion of nutrients and the accumulation of waste metabolic products. Overall, the growth pattern of the three isolates was similar in Marine Broth. Furthermore, the growth curves of our strains are typical of those observed for bacteria (Wang et al., 2015).

### Genome Analysis

The basic statistics of the assembled genomes of RO1, RO2 and RO3 are given in Supplementary Table 1 and in Rehman et al. (2020b). Briefly, the sizes of genomes were between 4.1 and 4.2 Mbp, with completeness more than 98%. The BLASTn analysis of 16S rRNA genes of the isolates was conducted against 16S rRNA sequences (bacteria and archaea) database (NCBI, March 29, 2020) (Altschul et al., 1997). These analyses showed that the best match for RO1 (92.3% identity, 98% coverage) and RO2 (99.9% identity, 100% coverage) was *Bacillus horikoshii* (NR_119070.1), whereas the best match for RO3 (99.4% identity, 97% coverage) was *Bacillus aquimaris* (NR_025241.1).

Taxonomic analysis using a single gene such as 16S rRNA gene fails to provide sufficient resolution at the species level. Consequently, Average Nucleotide Identity (ANI) is fast becoming a preferred method to classify the newly sequenced genomes. The bacteria belonging to the same species typically showed >=95% ANI values (Jain et al., 2018). We used Genome taxonomy Database Toolkit (GTDB-Tk v1.0.2) to classify each genome by placing it in a reference phylogenetic tree and calculating ANI values against the reference genomes (Chaumeil et al., 2019). The GTDB-Tk analysis showed that both RO1 (91% ANI) and RO2 (92.2% ANI) are closely related to *Bacillus* sp. CHD6a (GCF_001293645.1), while RO3 (83.1% ANI) is related of *B. aquimaris* (GCF_000935355.1) (Supplementary Table 2). The ANI values, less than 95%, suggest that the RO isolates may represent novel species belonging to the genus *Bacillus* (Rashid et al., 2015). Furthermore, it appears that RO1 and RO2 represent two distinct species, as ANI between them was 92.74%, whereas RO1 and RO3 showed 77.95% ANI, and RO2 and RO3 showed 78.1% ANI values.

**FIGURE 1:**
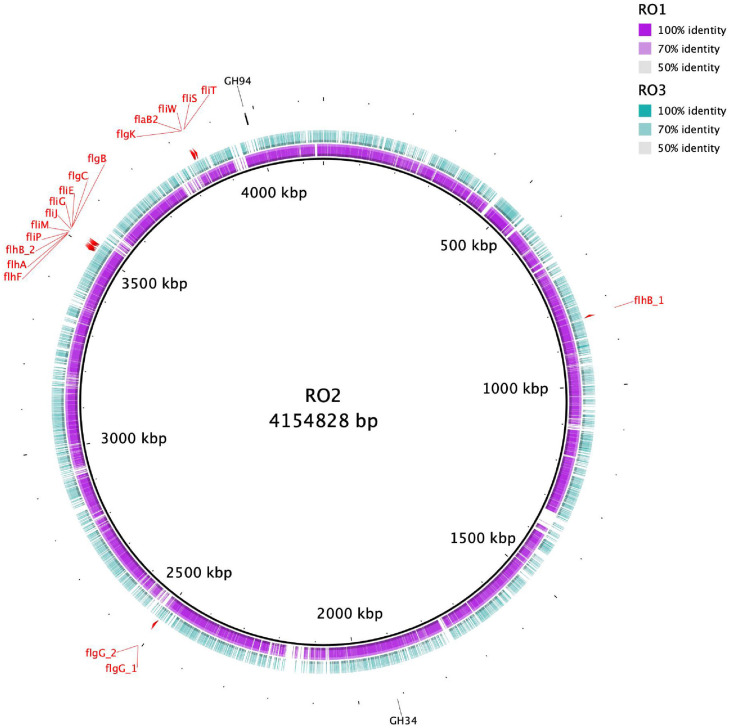
Circular representation of RO isolates genomes. The inner black circle represents the reference RO2 genome with genetic coordinates. The colored rings show RO1 and RO3 genomes, which were used as a query. RO1 genome is more homologous to RO2 compared to RO3. The genes involved in the biosynthesis of flagella in RO2 are shown. The CAZYmes that were detected only for RO2 are also indicated.

To identify proteins involved in carbohydrate metabolism a search against the dbCAN database was carried out, which showed that RO1, RO2, and RO3 carry 79, 84, and 76 carbohydrate-active enzymes (CAZymes), respectively (Supplementary Table 3). The detected CAZymes include Glycoside Hydrolases (GH), Carbohydrate Esterases (CE), Glycosyltransferases (GTs), as well as proteins with Carbohydrate-Binding Modules (CBM) (Supplementary Table 3). Most of the identified CAZymes families were shared between the RO isolates except GH35 and GH94, which were found only in RO2 (Supplementary Table 3 and Figure 1). The GH35 family includes B-galactosidases, exo-B-glucosaminidases, exo-B-1,4-galactanases, and B-1,3-galactosidases, whereas GH94 mainly act as phosphorylases^2^ (Lombard et al., 2014). It is interesting to note that RO2, whose EPS is smaller molecular weight compared to RO1 and RO3 (Table 1), contains two extra GHs. It remains to be determined if these GHs have any role in degradation and production of low molecular weight EPS by RO2.

**TABLE 1:** Elution range, molecular weight average, and polydispersity index (PDI) of EPS extracted from RO isolates.

**Sample**	**Content**	**Elution range (min)**	**Molecular weight average (MW)**	**PDI (MW/Mn^a^)**
RO1	Polysaccharides	12.4–13.7	201.3 KDa	1.1
	UV absorbing substances	14.2–16.5	8.9 KDa	1.7
		16.6–18	0.9 KDa	1.4
RO2	Polysaccharides	13.3–14.7	62.4 KDa	1.2
	UV absorbing substances	14.7–16.5	7.9 KDa	1.5
		16.6–18	0.9 KDa	1.4
RO3	Polysaccharides	12.1–13.9	213.1 KDa	1.3
	UV absorbing substances	14.1–17.9	2.7 KDa	3.1

*^*a*^Number average molecular weight.*

All the isolates contain YwqC, YwqD, and YwqE proteins encoded by *ywqCDEF* operon, which is implicated in the synthesis of capsular polysaccharides (Supplementary Table 4). The protein YwqD is a tyrosine kinase that phosphorylates YwqF and TuaD, and UDP-glucose dehydrogenases, which are the key enzymes required for capsular polysaccharide biosynthesis (Mijakovic et al., 2003). Studies show that the eps operon (*epsABCDEFGHIJKLMNO*) of *B. subtilis* synthesize major components of biofilm EPS (Vlamakis et al., 2013). EpsE, the best-studied protein encoded by eps operon is bifunctional; acting as glycosyltransferase and a molecular clutch inhibiting flagellar rotation (Blair et al., 2008). All the RO isolates possess proteins that show significant similarity to EpsE protein (Supplementary Table 4). These results show that RO isolates have the genetic potential to synthesize EPS components required for biofilm formation by the genus *Bacillus*, and that the polysaccharide component of EPS may be similar in composition to *B. subtilis* given the genetic similarities. However, further research is needed to confirm this suggestion.

The proteins encoded by *tapA-sipW-tasA* operon produce a major protein component of *B. subtilis* biofilms (Branda et al., 2006). The TasA forms amyloid fibers anchored to cell wall through TapA (Romero et al., 2010). SipW is a type I signal peptidase needed to process and release TasA and TapA to the extracellular milieu (Terra et al., 2012). Genome analyses reveal that only RO3 contain the genes that encode proteins similar to TasA, TapA, and SipW (Supplementary Table 4). The absence of TasA, TapA, and SipW similar proteins in RO1 and RO2 could result from incomplete genome sequences, as the genome sequences of RO1 and RO2 are 98.56% complete, whereas RO3 is 98.85% complete (Rehman et al., 2020b). Another possibility is that RO1 and RO2 produce functionally similar proteins with different primary sequences that could not be identified by blastp analysis.

Genome analysis showed that all the RO *Bacillus* strains carry genes, albeit different in number, involved in the biosynthesis of flagella (Figure 1). We detected 18 flagellar biosynthesis genes in the RO2 genome, and their organization in the genome is shown in Figure 1. Different flagellar biosynthesis genes found in the genomes of RO1, RO2, and RO3 are given in Supplementary Table 5. Bacterial flagella not only play a role in cellular motility but also take part in surface sensing and formation of biofilm (Belas, 2014). The RO membranes have different surface properties, such as hydrophobicity, surface roughness, and charge (Nguyen et al., 2012). Flagellar motion allows bacteria to overcome the hydrodynamic conditions and the repulsive forces due to surface chemistry (Lemon et al., 2007). Once attached, the *Bacillus* strains can produce EPS and activate other genes involved in the formation and further development of biofilm (Lee et al., 2010).

Previously, we used high-throughput sequencing to identify the composition and functional potential of the microbial community on the biofouled RO membranes (Rehman et al., 2019). We found that *Firmicutes* (to which genus *Bacillus* belongs) constitute the fourth most abundant Phyla detected on biofouled RO membranes. However, the relative abundance of *Bacillus* in the context of biofouling may be irrelevant. A previous study on the biofouling of the RO membrane using pure cultures of *Bacillus* showed that it is not the cell number of *Bacillus*, but their EPS and activation of the specific genes that determine the extent of biofouling (Lee et al., 2010).

### RO Isolates Form Less Biofilm Under Laboratory Conditions

**FIGURE 2:**
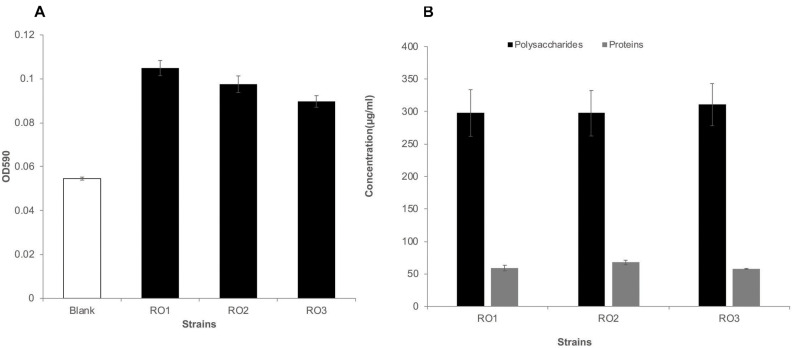
Biofilm formation and EPS production by RO isolates. Biofilm formation by the RO membrane isolates (RO1, RO2, and RO3) on polystyrene surface after 48 h, given as optical density at 590 nm (*y*-axis) **(A)**. Blank represents marine broth without inoculation. The height of bars shows the average of three biological replicates, while error bars represent standard deviation. Student’s *t*-test showed no significant difference in biofilm formation by the three isolates (*p* > 0.05). The amount of polysaccharides (black) and proteins (gray) produced by the RO isolates (RO1, RO2, and RO3) was similar **(B)**. The quantity of polysaccharide and proteins is given as μg/ml of culture media along the *y*-axis. The height of bars shows the average of three independent measurements, while error bars show standard deviation. The amount of polysaccharides was approximately five times higher than the amount of proteins.

After 24 h of incubation, biofilm formation was not detected for any isolate on polystyrene plates. It is possible that the RO isolates (Gram-positive *Bacillus*) were slow biofilm formers compared to biofilm forming *Pseudomonas aeruginosa* studied in our lab (Rehman and Leiknes, 2018), or that the laboratory conditions and culture media were not suitable for biofilm formation by these bacteria. Furthermore, the negative charges on *Bacillus* spp. (Malanovic and Lohner, 2016) and polystyrene surfaces (tissue culture treatment) may have prevented the initial attachment of bacteria to the surface. Indeed, Pang et al. (2005) have demonstrated that bacteria with higher negatively charged surfaces form less biofilm (Pang et al., 2005). Nonetheless, after 48 h of incubation, we observed biofilm formation for all the isolates, albeit in low amounts (Figure 2A). The formation of a surface-conditioning film most likely allowed the bacteria to overcome the electrostatic repulsion and attach to the surface (Lorite et al., 2011). We observed a slightly higher biofilm formation by these isolates on glass surface compared to polystyrene after 48 h (Supplementary Figure 3), which can be attributed to the different surface properties of glass and polystyrene. Similar to our results, Pang et al. (2005) have shown that some of their RO isolates yielded low biomass in a biofilm formation assay (Pang et al., 2005). There was no statistically significant difference in biofilm formed by the three isolates (*p* > 0.05).

Note that the bacteria isolated in this study represent only a fraction of the bacterial diversity on the RO membrane (Rehman et al., 2019). The microbes in RO elements are exposed to harsh conditions, such as high pressure, salinity, and shear force (Nagaraj et al., 2017a), and such conditions may be selective for microbes that have superior attachment and ability to survive under these conditions. It is not easier to replicate these harsh conditions in the lab, making it challenging to capture bacterial diversity through cultivation-based methods. Therefore, it is important to study the phenotypic and chemical characteristics of cultivable bacteria frequently detected in the RO system (Belila et al., 2016; Nagaraj et al., 2017b). Furthermore, *Bacillus* are chlorine resistant, which allows them to survive the pretreatment and deposit on the membrane, contributing to biofouling and flux decline (Bhojani et al., 2018).

### RO Isolates EPS Contain a High Amount of Polysaccharides

In this study we extracted and analyzed the secreted fraction of EPS, which has a higher binding capacity (Kim et al., 2006). The amount of proteins and polysaccharides produced by all three isolates growing in biofilm mode was very similar (Figure 2B). The DNA was below the detection limit in the extracted EPS. Our results are consistent with those reported for acid mine drainage biofilms and EPS isolated from biofilm-forming marine bacteria (Jiao et al., 2010; Siddik and Satheesh, 2019), where a higher carbohydrate-to-protein ratio was reported. Furthermore, we have previously shown that the carbohydrate-to-protein ratio increases as the biofilm grows older (Matar et al., 2016). Jiao et al. (2010) suggested that the low protein content in the EPS of biofilms could be attributed to protein degradation by extracellular proteases and by harsh conditions. Further, studies have shown that marine bacterial isolates can produce extracellular proteases (Fulzele et al., 2011), which can potentially degrade extracellular proteins.

### RO Isolates Produce High Molecular Weight EPS

The molecular weight distribution of the EPS extracted from the three isolates was determined using size exclusion chromatography. The refractive index (RI) and UV chromatogram of the EPS samples were similar to those recently reported (Liu, 2018; Supplementary Figure 4). The RI chromatogram shows that the EPS from RO1, RO2, and RO3 were eluted as two size groups by the HPSEC column (Table 1 and Supplementary Figure 4). The first group (peak 1) of RO1 was eluted in the range of 12.4–13.7 min, and the second group (peak 2 and 3) was eluted between 14.2 and 18.0 min (Table 1). For RO2, the first group (peak 1) of the EPS components was eluted in the range 13.3–14.7 min, and the second group (peak 2 and 3) was eluted between 14.7 and 18.0 min (Table 1). For RO3, the first peak was eluted between 12.1 and 13.9 min and the second peak between 14.1 and 17.9 min (Table 1).

The size exclusion chromatography (SEC) column separates molecules based on their hydrodynamic volumes with larger molecules elute faster than smaller molecules. The first fraction (peak 1) of the EPS, which eluted faster, showed no UV absorbance in all the samples (Supplementary Figure 4). We identified this higher molecular weight non-UV absorbing fraction of EPS as polysaccharides (Alasonat and Slaveykova, 2012). The second group (peak 2 and 3) of the EPS components, which absorbed UV, was most likely proteinaceous substances as no DNA was detected in the EPS samples. The peaks represent refractive index (RI), which depends on the molar concentration and properties of the solute in a multi-component solution (Zhu et al., 2016). Therefore, the peak size may not reflect the amount of polysaccharide and protein. The peaks that were outside the calibration range of the column were not analyzed (Supplementary Figure 4).

The average molecular weights of extracellular polymers produced by RO1, RO2, and RO3 were 201.3, 62.4, and 213.1 KDa, respectively. The molecular weight of polysaccharides from strain RO2 was smaller than RO1 and RO3 (Table 1). However, the molecular weight of the EPS components from all the isolates was within the range described in previous studies (Brian-Jaisson et al., 2016; Liu, 2018). We observed that the polydispersity index (PD) of the proteinaceous substances was slightly higher than the PD of the polysaccharides, possibly because formaldehyde and NaOH can destroy disulfide bonds in glycoproteins, which could lead to higher polydispersity (Alasonat and Slaveykova, 2012).

The high molecular weight exopolysaccharides produced by RO isolates may serve as a scaffold for the attachment of proteins, lipids, and nucleic acids, which may cumulatively form biofilm matrix (Bales et al., 2013). Studies have shown that high molecular weight organic matter is responsible for fouling of NF membranes (Nilson and DiGiano, 1996). The physical properties of EPS produced by the RO isolates suggest that they can attach to and condition the surfaces for subsequent biofilm formation.

### Identification of Functional Groups Associated With the Fouling Potential of EPS

FTIR analysis was performed to identify the functional groups associated with the EPS polymers. The peaks were assigned to functional groups based on previous studies (Jiao et al., 2010; Brian-Jaisson et al., 2016; Maddela et al., 2018). Overall, the FTIR spectra of the isolates were very similar, indicating that the chemical composition of the EPS produced by these isolates was also very similar.

**FIGURE 3:**
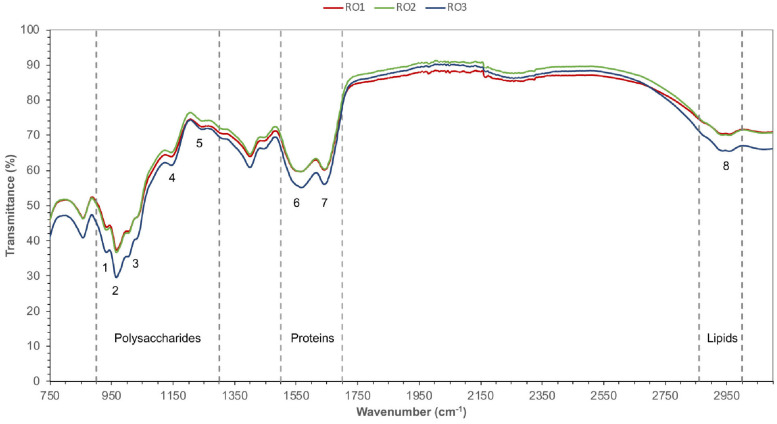
FTIR spectra of EPS. Different functional groups (1–8) belonging to the expected type of chemical compounds (polysaccharides, proteins, and lipids) were identified based on characteristic IR bands. Transmittance (%) is given along the y-axis, while the wavelength of infrared radiation is indicated along the x-axis. Peak 1 and 2 (α-1,4 glycosidic linkage), peak 3 (uronic acids), peak 4 (*O*-acetyl group), peak 5 (carboxylic group), peak 6 (amide II), peak 7 (amide I), peak 8 (lipids).

The FTIR spectra in the range 900–1,300 cm^–1^ are characteristic of functional groups in polysaccharides and nucleic acids (Brian-Jaisson et al., 2016). However, DNA was not detected in the EPS samples; thus, spectra in this range most probably corresponded to polysaccharides (Figure 3). FTIR analysis of the EPS from all the isolates showed the presence of α-1,4 glycosidic linkage (peak 1 and 2), uronic acids (peak 3), *O*-acetyl group (peak 4), and carboxylic group (peak 5) (Figure 3; Tang et al., 2017; Maddela et al., 2018). Jiao et al. (2010) previously suggested that the proteins show FTIR spectra in the range of 1,500–1,700 cm^–1^ (Jiao et al., 2010). In this range, peak 6 corresponds to stretching vibrations of C = O and C-N [amide (II)] and peak 7 corresponds to C = O and C-N [amide (I)] groups commonly found in the peptide bonds of proteins (Alasonat and Slaveykova, 2012; Tang et al., 2017). Lastly, peak 8 represents C-H vibrational stretching of aliphatic hydrocarbons, which is indicative of lipids (Figure 3; Jiao et al., 2010; Tang et al., 2017).

In a recent study, peak 1, 2, and 6 were correlated with the fouling potential of pure cultures of bacteria (Maddela et al., 2018). Furthermore, the authors suggested that EPS of high fouling bacterial strains contained uronic acids (peak 3) and *O*-acetyl groups (peak 4), which imparted gelling properties to EPS and enhanced attachment of bacteria to the membrane.

Overall, the FTIR spectra confirmed the presence of proteins, polysaccharides, and lipids in the EPS produced by the RO isolates. The results show that the *Bacillus* are capable of producing diverse biological molecules with varying chemical nature and charge. These molecules may facilitate biofilm formation by complex communities of microbes on the RO membrane.

### Higher Abundance of Aliphatics of EPS

**FIGURE 4:**
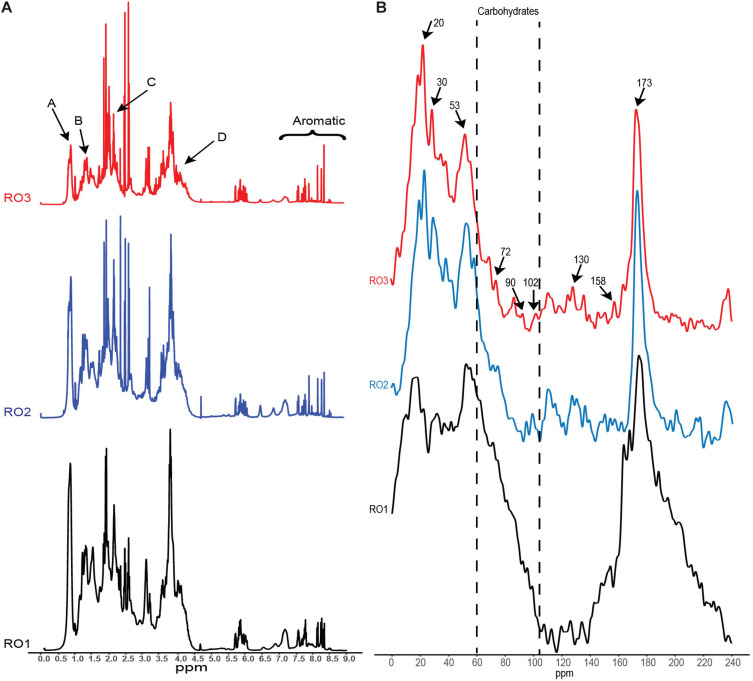
NMR analysis of the EPS. **(A)** ^1^H NMR spectra showing peaks (A, B, C, D) corresponding to different functional groups associated with specific chemical compounds. **(B)** Solid-state ^13^C NMR spectra revealing chemical structures in the EPS. Arrows indicate the chemical shifts that were identified based on (Khan et al., 2014). Y-axis (not shown) represents signal intensity in arbitrary units.

^1^H NMR analysis was carried out to further explore the chemical composition of EPS obtained from the RO isolates (Figure 4A). The three EPS samples showed well-resolved peaks. The relative abundance of the five ^1^H chemical shifts regions was calculated, which were assigned based on (Table 2; Hertkorn et al., 2013). These results reveal small differences in the relative abundance of different structural categories of organic H between EPS of the RO isolates. However, these differences may not be significant as suggested by previous studies (Hertkorn et al., 2013; Fox et al., 2018).

**TABLE 2:** Abundance (%) of major fractions of ^1^H chemical shifts assigned as previously (Hertkorn et al., 2013; Fox et al., 2018).

	**^δ^H (ppm)**				
**Samples**	**10.0–7.0**	**7.0–4.9**	**4.1–3.1**	**3.1–1.9**	**1.9–0.2**
	**Aromatic**	**Olefinic^a^**	**HC-O^b^**	**Functionalized^c^**	**Aliphatic^d^**
RO1	4.9	3.1	24.6	26.9	33.9
RO2	4.6	3.1	25.7	30	31
RO3	4.6	4	29	29.7	25.6

*^*a*^Protons assigned to HC = C groups in olefinic compounds.*

*^*b*^Protons in this range assigned to HC-O (single-oxygenated carbon and hydrogen) associated with carbohydrate.*

*^*c*^Protons associated with carboxyl-rich alicyclic molecules, amino sugars and proteins.*

*^*d*^Protons in aliphatics with heteroatoms 4 or more bonds away.*

Results based on the five ^1^H chemical shifts showed that EPS from the RO isolates was similar to EPS of dissolved organic matter obtained from various marine sources (Hertkorn et al., 2013; Fox et al., 2018). Our EPS samples showed a higher abundance of aliphatics (Table 2), similar to what has been reported for dissolved organic matter from reverse osmosis-electrodialysis system (RO-ED) (Table 2; Koprivnjak et al., 2009). The aliphatics were the most abundant structural category of organic H identified for RO1 and RO2 (Table 2). These results suggest that hydrophobic compounds constitute a significant proportion of organic material deposited on RO membranes. Also, the abundance of HC-O (singly-oxygenated hydrogen and carbon) chemical shifts (ascribed to carbohydrates) in the EPS of RO isolates and organic matter from RO-ED (Koprivnjak et al., 2009) was similarly high. However, the abundance of HC-O was found to be lower in organic matter from other marine sources (Fox et al., 2018). The higher abundance of HC-O in RO-ED and also in our samples indicates that carbohydrates have a higher binding capacity to the RO membranes. However, experiments using purified polysaccharides and their ability to bind to RO membrane are needed to confirm this.

Furthermore, we identified proton chemical shifts indicating CH_3_ groups (Peak A, 0.85 ppm), which were previously reported for exopolymers obtained from bacteria but not from algae (Figure 4A; Li et al., 2015). Similarly, CH_2_ groups (Peak B, 1.25 ppm) associated with cutins, lipids, and waxes were also detected (Figure 3A; Simpson et al., 2007). We also observed proton resonances (Peak C, 2.1 ppm) indicating methyl groups of *N*-acetyl amino sugars (Figure 4A; Fox et al., 2018). Resonances corresponding to *N*-acetylated amino sugars have been reported for high molecular weight dissolved organic matter in surface seawater and semi-labile polysaccharides (Hertkorn et al., 2006; Abdulla et al., 2013). The acylated polysaccharides detected in the saline environment are more stable than their non-acetylated counterparts (Abdulla et al., 2010; Fox et al., 2018). Lastly, we detected chemical shifts (Peak D, 4.1 ppm) indicating fucosylated compounds in the EPS (Figure 4A). These compounds are produced by marine bacteria and play a role in the attachment of barnacles on different surfaces (Siddik and Satheesh, 2019), suggesting their role in surface conditioning.

Furthermore, the peaks in the aromatic area at 7.1 and 8.2 ppm corresponded to the aromatic amino acid side chain and amides in peptides, respectively, indicating presence of proteins and its hydrophobic nature. Proteins constitute an important component of transparent exopolymers (TEP), which are significant contributors to surface biofouling (Villacorte et al., 2013; Li et al., 2015). Based on the ^1^H NMR analyses, it appears that the EPS produced by the RO isolates has chemical features similar to that of organic matter found in marine environments. Furthermore, it appears that EPS is structurally stable and suited for harsh environments.

### EPS Carbohydrates Are Mainly Linked by α-Glycosidic Bonds

Solid-state ^13^C NMR spectroscopy was conducted to identify the different functional groups associated with EPS. The ^13^C carbon atoms resonate at different frequencies depending on the electronic structure of the immediate environment. Aliphatic carbons resonate in the frequency range of 0–40 ppm, while secondary alcohols of carbohydrates and carbon atoms of glycoside bonds resonate in the frequency range of 60–90 and 95–106 ppm, respectively (Jiao et al., 2010). The resonance frequency of glycosidic carbon is also indicative of their linkage, i.e., alpha or beta oxygen linkages. Carbon atoms forming linkages to alpha oxygen resonate in the range 95–103 ppm, while linkages to beta oxygen resonate in the range 103–106 ppm.

As shown in Figure 4B, the peak observed at 20 ppm indicates methyl carbon (Khan et al., 2014), while the peak at 30 ppm indicates methylene groups in long-chain hydrocarbons (Metzger et al., 2009; Jiao et al., 2010), suggesting the presence of hydrophobic compounds in EPS. These hydrophobic compounds may represent lipids that originate from phospholipids and lipopolysaccharides of the bacterial cell membrane (Metzger et al., 2009). Recently, it has been shown that lipids constitute a considerable fraction of the organic matter deposited on the RO membrane treating seawater (Khan et al., 2014). In line with previous studies such as (Metzger et al., 2009), it appears that EPS produced by the bacterial isolates in this study contain large amounts of lipids. The presence of lipids in the EPS samples was also confirmed by ^1^H NMR analysis (Table 2).

The NMR spectra confirmed the presence of secondary alcohols and revealed the type of glycoside bonds linking the monomers in the bacterial isolates. We detected a peak at 72 ppm, indicative of secondary alcohol carbon, in all the EPS samples (Figure 4B), albeit much lower in RO1 (Khan et al., 2013). The peaks at 90 and 102 ppm indicated that carbohydrates in all the samples contain α-glycoside bonds (Figure 4B). However, the peak at 102 ppm is small in RO2 and RO3 compared to RO1. It is not clear if the small peak for α-glycosidic bonds in RO2 can account for low molecular weight EPS produced by this bacteria (Table 1). FTIR analysis also confirmed the presence of α-1,4 glycoside linkage in all the EPS samples (Figure 4). The peaks at 130 and 158 ppm were indicative of proteins containing hydroxy aromatic amino acids, such as tyrosine (Figure 4B; Khan et al., 2013). These peaks were detected in all the EPS samples from RO isolates. A sharp peak at 173 ppm indicated the presence of an amide carbon, suggesting the presence of proteins in the sample (Figure 4B; Jiao et al., 2010).

The ^13^C NMR spectra of EPS harvested from the RO membrane bacterial isolates show many resonances that have been previously detected in foulants isolated from RO membranes used for treating seawater (Khan et al., 2014, 2015). Based on NMR spectra, Khan et al. (2013) argued that the foulants on RO membranes mainly originated from bacterial cells. Our results support this claim, where the ^13^C NMR spectra of EPS produced by bacteria isolated from the biofouled RO membrane in this work bear similarity to foulants on the RO membranes (Figure 4B). Therefore, EPS produced by our isolates can be used in studies investigating the role of EPS in membrane conditioning, biofilm initiation and flux decline of RO membranes.

## Conclusion

This study provides valuable information on the physicochemical properties of EPS produced by bacteria that thrive on biofouled RO membranes. We acknowledge that biofouling is caused by diverse microbes, which produce heterogenous EPS. Nevertheless, EPS investigated in this study is a suitable representative example of typical EPS found on biofouled RO membranes. Furthermore, culture-based and culture-independent studies have demonstrated that *Bacilli* are ubiquitous in RO systems. Therefore, *Bacilli* should be investigated with interest in the context of RO biofouling.

**FIGURE 5:**
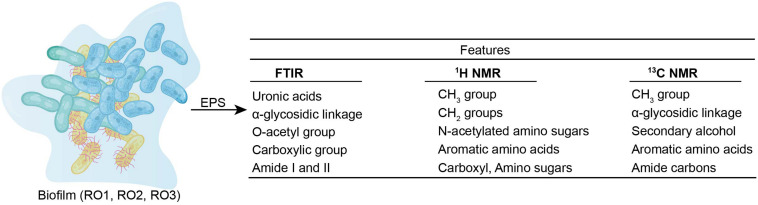
Chemical features of EPS (RO1, RO2, and RO3) as identified by FTIR, 649 1 H NMR, and 13 650 C NMR are given.

This study provides an in-depth chemical characterization of EPS produced by *Bacillus* (Figure 5). We found that the EPS contained a high abundance of hydrophobic substances that are linked to significant flux decline in membrane processes. We also identified structural features (glycosidic bonds, *O*-acetyl functional groups, and amine sugars), which are essential determinants of the strength and the biofouling potential of EPS. Furthermore, various genes involved in biofilm and EPS formation were identified. However, this remains to be determined if genetic similarity translates into EPS compositional similarity.

The findings reported in this study can be used to explore future biofouling control strategies, either by improving pretreatment methods or interfering with the deposition of organic substances and microbes on membranes. Although most pretreatment methods effectively remove contaminants in feedwater. However, any microbes that manage to reach the membrane can potentially grow, produce EPS, and eventually form biofilms. Modification of the membrane surface to prevent the adhesion of EPS molecules should be explored further. Furthermore, the features of EPS observed in this study could guide the selection of EPS-degrading enzymes, such as glycoside hydrolases, lipases, and proteinases as potential biofilm control strategies.

## Data Availability Statement

The datasets presented in this study can be found in online repositories. The names of the repository/repositories and accession number(s) can be found in the article/Supplementary Material.

## Author Contributions

ZR conceived and performed the experiments, and wrote the manuscript. PS and JV reviewed and edited the manuscript. All authors contributed to the article and approved the submitted version.

## Conflict of Interest

The authors declare that the research was conducted in the absence of any commercial or financial relationships that could be construed as a potential conflict of interest.

## Footnotes

1 http://bcb.unl.edu/dbCAN2/blast.php

2 http://www.cazy.org/
