# Supplementary material for: Physicochemical Properties of Extracellular Polymeric Substances Produced by Three Bacterial Isolates From Biofouled Reverse Osmosis Membranes
Source: Front Microbiol. 2021 Jul 13;12:668761. doi: 10.3389/fmicb.2021.668761 (PMC8328090; doi:10.3389/fmicb.2021.668761)
Supplement: Supplementary file 1 [file Table_1.docx]

| **Sample name** | **Assembly length (bp)** | **No. of contigs** | **GC (%)** | **Contigs N50** | **Completeness (%)** | **Coverage** |
| --- | --- | --- | --- | --- | --- | --- |
| RO1 | 4224738 | 92 | 40 | 92363 | 98.85 | 217 |
| RO2 | 4154828 | 57 | 40 | 118722 | 98.56 | 268 |
| RO3 | 4223915 | 34 | 43 | 226369 | 98.56 | 238 |

Table S1. Sample and sequencing statistics
