# Supplementary material for: Physicochemical Properties of Extracellular Polymeric Substances Produced by Three Bacterial Isolates From Biofouled Reverse Osmosis Membranes
Source: Front Microbiol. 2021 Jul 13;12:668761. doi: 10.3389/fmicb.2021.668761 (PMC8328090; doi:10.3389/fmicb.2021.668761)
Supplement: Supplementary file 3 [file Table_3.docx]

Table S3: List of CAZYmes found in the genomes of RO1, RO2, and RO3. The letters AA, CBM, CE, GH, and GT indicate class of CAZYme, that is, Auxiliary Activities, Carbohydrate Binding Modules, Carbohydrate Estrases, Glycoside Hydrolases, Glycoside Transferases. The enzyme class is followed by number that reflects the family, and the number followed by “_” indicates subfamily. Protein containing multiple domains belonging to different classes of CAZYmes are indicated by “+” sign between each class.

| **CAZY families** | **RO1** | **RO2** | **RO3** |
| --- | --- | --- | --- |
| AA2+AA2 | 1 | 1 | 1 |
| AA4 | 1 | 1 | 1 |
| AA6 | 1 | 1 | 1 |
| CBM34+GH13_20 | 1 | 1 | 1 |
| CBM41+GH13_41+CBM41+CBM41+GH13_12 | 1 | 1 | 1 |
| CBM50 | 2 | 2 | 2 |
| CBM68+GH13_14 | 1 | 1 | 1 |
| CE1 | 1 | 2 | 1 |
| CE10 | 3 | 3 | 3 |
| CE14 | 3 | 3 | 3 |
| CE4 | 4 | 4 | 4 |
| CE9 | 1 | 1 | 1 |
| GH1 | 1 | 1 | 1 |
| GH109 | 3 | 4 | 3 |
| GH13_1 | 1 | 1 | 1 |
| GH13_29 | 1 | 1 | 1 |
| GH13_31 | 3 | 3 | 3 |
| GH13_5 | 1 | 1 | 1 |
| GH13_9 | 1 | 1 | 1 |
| GH144 | 2 | 2 | 2 |
| GH16+CBM4+CBM4+CBM4+CBM4 | 1 | 1 | 1 |
| GH18 | 2 | 2 | 2 |
| GH23 | 1 | 1 | 1 |
| GH3 | 1 | 2 | 1 |
| GH30_1 | 1 | 1 | 1 |
| GH31 | 1 | 1 | 1 |
| GH32 | 1 | 1 | 1 |
| **GH35** |  | 1 |  |
| GH73 | 1 | 1 | 1 |
| **GH94** |  | 1 |  |
| GT2_Glyco_tranf_2_3 | 2 | 2 | 2 |
| GT2_Glycos_transf_2 | 10 | 10 | 9 |
| GT28 | 2 | 2 | 2 |
| GT32 | 1 | 1 | 1 |
| GT35 | 1 | 1 | 1 |
| GT4 | 15 | 15 | 13 |
| GT5 | 1 | 1 | 1 |
| GT51 | 5 | 5 | 5 |
