# Supplementary material for: Physicochemical Properties of Extracellular Polymeric Substances Produced by Three Bacterial Isolates From Biofouled Reverse Osmosis Membranes
Source: Front Microbiol. 2021 Jul 13;12:668761. doi: 10.3389/fmicb.2021.668761 (PMC8328090; doi:10.3389/fmicb.2021.668761)
Supplement: Supplementary file 4 [file Table_4.DOCX]

Table S4: Genes detected in RO isolates contributing to polysaccharides and protein components of EPS. The locus ID of proteins (showing similarity to EPS producing proteins) is given according to NCBI record. Percentage identity and E-values as computed by blastp are also given.

|  | **RO1** | | | **RO2** | | | **RO3** | | |
| --- | --- | --- | --- | --- | --- | --- | --- | --- | --- |
|  | Locus ID | Identity | E-value | Locus ID | Identity | E-value | Locus ID | Identity | E-value |
| TasA (AFO85459.1) | NF | NA | NA | NF | NA | NA | NMH67630.1 | 32% | 1e-26 |
| TapA (QVK13223.1) | NF | NA | NA | NF | NA | NA | NMH70563.1 | 26% | 7e-06 |
| SipW (QJD04760.1) | NF | NA | NA | NF | NA | NA | NMH67629.1 | 50% | 1e-48 |
| YwqF (CAB07444.1) | NLP50752.1 | 32% | 3e-60 | NMH75166.1 | 50% | 9e-154 | NMH67693.1 | 53% | 4e-154 |
| YwqC (CAB07455.1) | NLP51834.1 | 53% | 1e-80 | NMH73895.1 | 51% | 2e-82 | NMH70542.1 | 55% | 2e-85 |
| YwqD (CAB07457.1) | NLP51835.1 | 58% | 6e-92 | NMH73896.1 | 60% | 3e-94 | NMH68921.1 | 34% | 1e-39 |
| YwqE (CAB07456.1) | NLP52728.1 | 54% | 8e-97 | NMH7517.1 | 54% | 6e-93 | NMH70540.1 | 54% | 3e-98 |
| EpsE (ACR78276.1) | NLP49428.1 | 28% | 4e-09 | NMH75169.1 | 35% | 3e-32 | NMH67694.1 | 31% | 3e-08 |

NF= Not found

NA= Not applicable

First column represents target proteins with their NCBI reference number given in brackets.
