# Supplementary material for: Physicochemical Properties of Extracellular Polymeric Substances Produced by Three Bacterial Isolates From Biofouled Reverse Osmosis Membranes
Source: Front Microbiol. 2021 Jul 13;12:668761. doi: 10.3389/fmicb.2021.668761 (PMC8328090; doi:10.3389/fmicb.2021.668761)
Supplement: Supplementary file 5 [file Table_5.docx]

| **RO1** | **RO2** | **RO3** |
| --- | --- | --- |
| Flagellar secretion chaperone FliS | Flagellar secretion chaperone FliS | Flagellar secretion chaperone FliS |
| Flagellar motor switch protein FliM | Flagellar protein FliT | Flagellar motor switch protein FliM |
| Flagellar motor switch protein FliG | Flagellar motor switch protein FliM | Flagellar motor switch protein FliG |
| Flagellar hook-basal body complex protein FliE | Flagellar motor switch protein FliG | Flagellar hook-basal body complex protein FliE |
| Flagellar FliJ protein | Flagellar hook-basal body complex protein FliE | Flagellar hook-associated protein 3 |
| Flagellar biosynthetic protein FliP | Flagellar hook-associated protein 1 | Flagellar hook-associated protein 2 |
| Flagellar biosynthetic protein FlhB | Flagellar FliJ protein | Flagellar hook-associated protein 1 |
| Flagellar biosynthetic protein FlhB | Flagellar filament 31.3 kDa core protein | Flagellar hook protein FlgE |
| Flagellar biosynthesis protein FlhF | Flagellar biosynthetic protein FliP | Flagellar FliJ protein |
| Flagellar biosynthesis protein FlhA | Flagellar biosynthetic protein FlhB | Flagellar biosynthetic protein FliP |
| Flagellar basal-body rod protein FlgG | Flagellar biosynthesis protein FlhF | Flagellar biosynthetic protein FlhB |
| Flagellar basal-body rod protein FlgG | Flagellar biosynthesis protein FlhA | Flagellar biosynthesis protein FlhF |
| Flagellar basal-body rod protein FlgC | Flagellar basal-body rod protein FlgG | Flagellar biosynthesis protein FlhA |
| Flagellar basal body rod protein FlgB | Flagellar basal-body rod protein FlgC | Flagellar basal-body rod protein FlgG |
| Flagellar assembly factor FliW | Flagellar basal body rod protein FlgB | Flagellar basal-body rod protein FlgF |
|  | Flagellar assembly factor FliW | Flagellar basal-body rod protein FlgC |
|  |  | Flagellar basal body rod protein FlgB |
|  |  | Flagellar assembly factor FliW |
|  |  |  |

Table S5: Flagellar biosynthesis genes detected in RO1, RO2, and RO3 genomes.
