# Supplementary material for: Physicochemical Properties of Extracellular Polymeric Substances Produced by Three Bacterial Isolates From Biofouled Reverse Osmosis Membranes
Source: Front Microbiol. 2021 Jul 13;12:668761. doi: 10.3389/fmicb.2021.668761 (PMC8328090; doi:10.3389/fmicb.2021.668761)
Supplement: Supplementary file 6 [file Image_1.pdf]

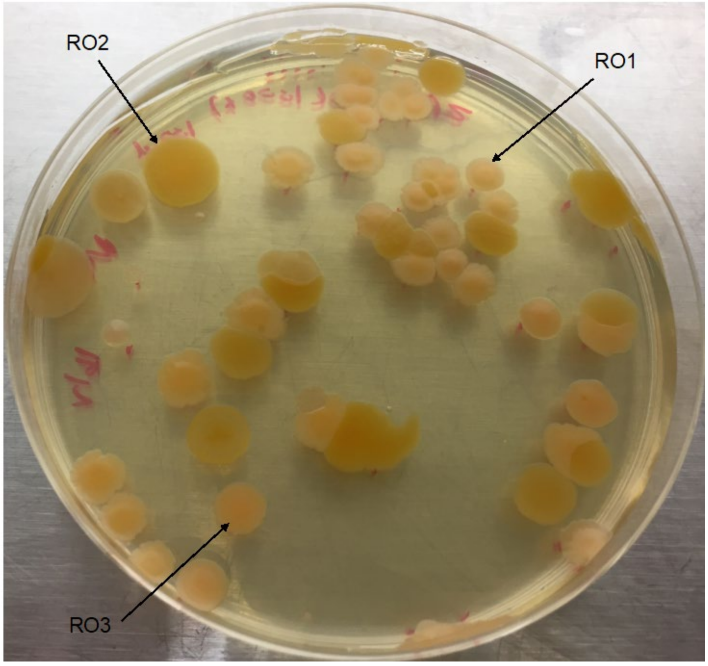

Supplementary figure 1. Biomass from the biofouled RO membrane was dissolved in PBS and spread on Marine Agar plates. Bacterial colonies with different phenotypes were selected (as shown) and purified for further analyses.
