# Supplementary material for: Physicochemical Properties of Extracellular Polymeric Substances Produced by Three Bacterial Isolates From Biofouled Reverse Osmosis Membranes
Source: Front Microbiol. 2021 Jul 13;12:668761. doi: 10.3389/fmicb.2021.668761 (PMC8328090; doi:10.3389/fmicb.2021.668761)
Supplement: Supplementary file 7 [file Image_2.pdf]

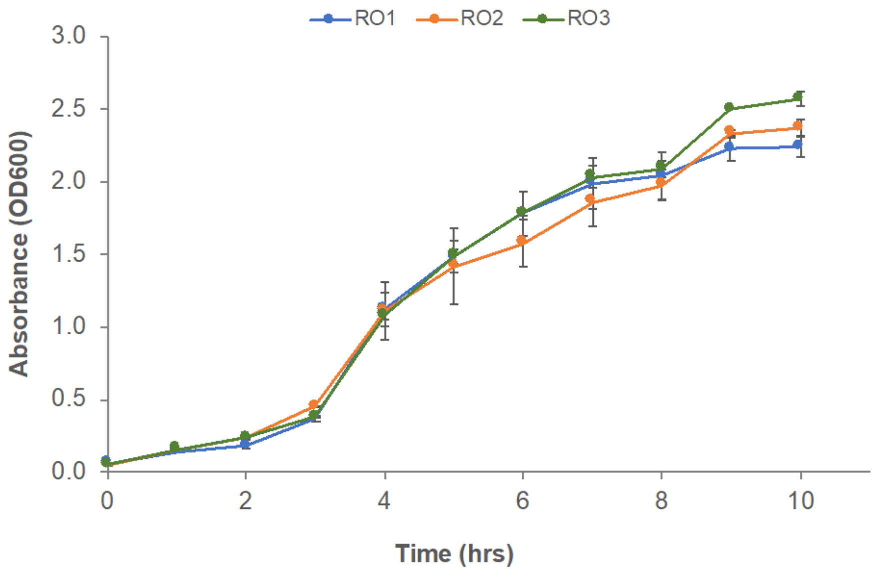


Supplementary figure 2. Growth curve of three RO membrane isolates in Marine Broth. Optical density at 600nm (y-axis) was measured every hour for ten hours (x-axis).
