# Supplementary material for: Physicochemical Properties of Extracellular Polymeric Substances Produced by Three Bacterial Isolates From Biofouled Reverse Osmosis Membranes
Source: Front Microbiol. 2021 Jul 13;12:668761. doi: 10.3389/fmicb.2021.668761 (PMC8328090; doi:10.3389/fmicb.2021.668761)
Supplement: Supplementary file 8 [file Image_3.pdf]

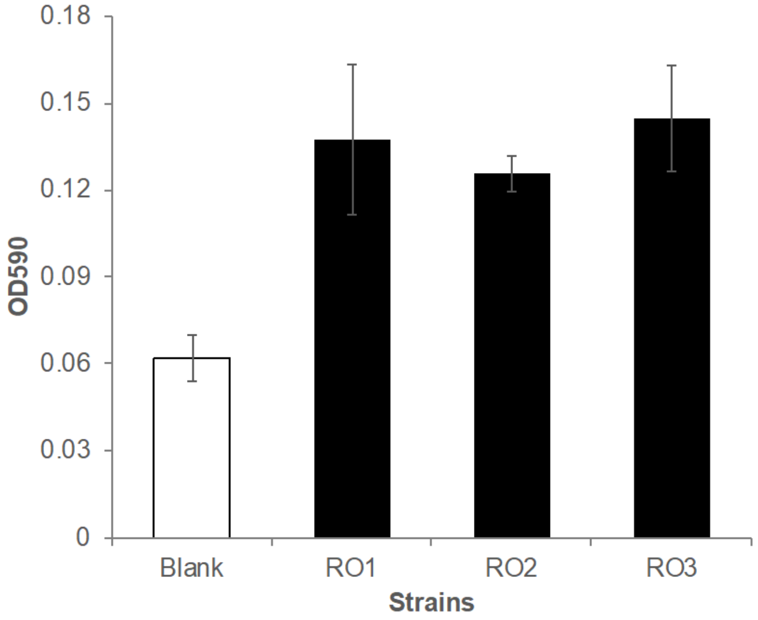

Supplementary figure 3. Biofilm formation by RO isolates. Biofilm formation by the RO isolates (RO1, RO2, and RO3 on a glass surface after 48 hrs. Blank represents marine broth without inoculation. The height of bars shows the mean of three independent replicates, while error bars represent standard deviation. Student's t-test showed no significant difference in biofilm formation by the three isolates (p-value > 0.05).
