# Supplementary material for: Physicochemical Properties of Extracellular Polymeric Substances Produced by Three Bacterial Isolates From Biofouled Reverse Osmosis Membranes
Source: Front Microbiol. 2021 Jul 13;12:668761. doi: 10.3389/fmicb.2021.668761 (PMC8328090; doi:10.3389/fmicb.2021.668761)
Supplement: Supplementary file 9 [file Image_4.pdf]

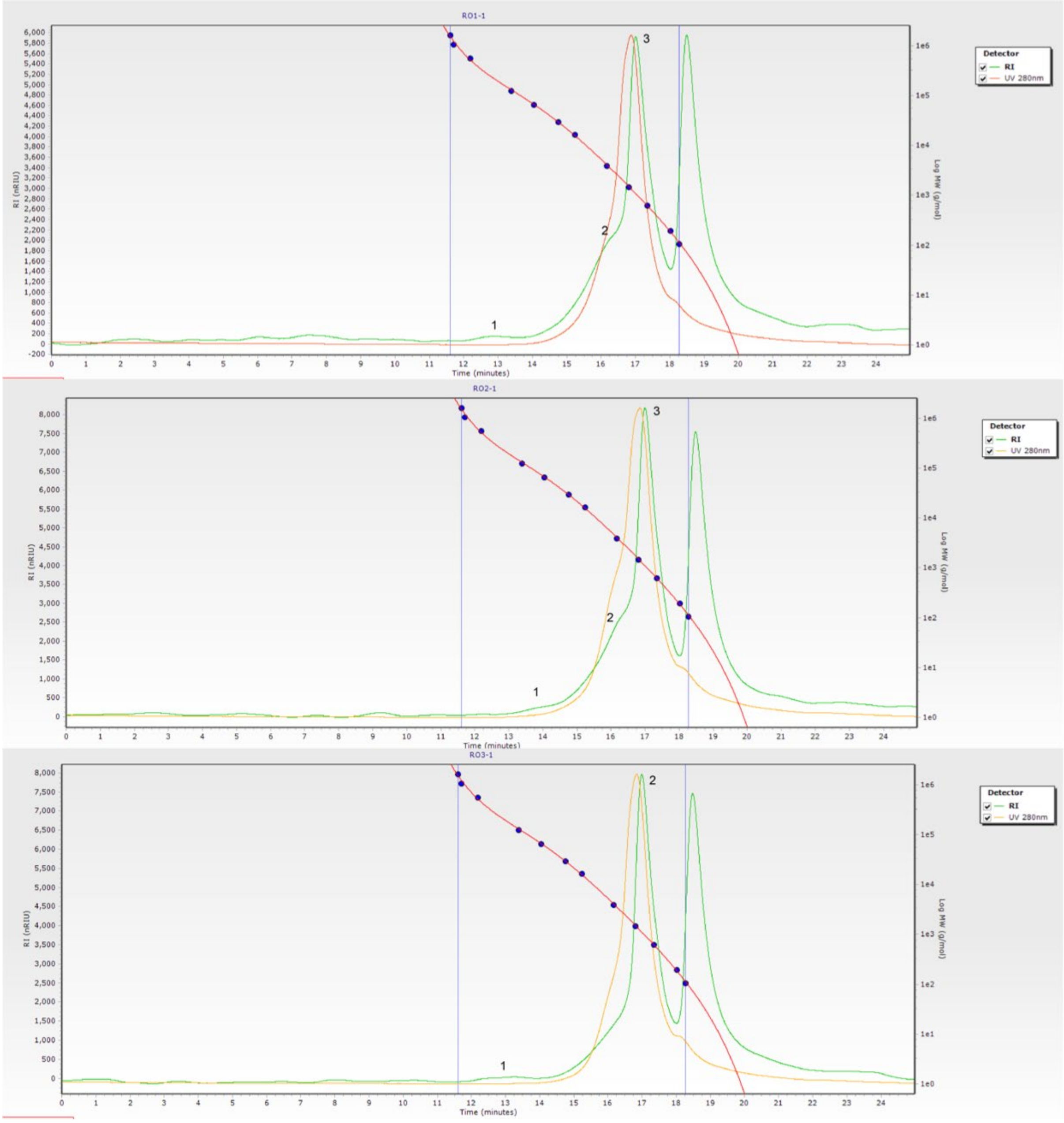

Supplementary figure 4. Chromatograms of refractive index and UV absorbance by EPS extracted from RO1(top), RO2(middle), and RO3(lower). Numbers (1, 2, 3) represent the peaks that were integrated for quantification. The red line is the calibration curve, and the blue dots represent the calibration points.
